# Cytoarchitecture of Breast Cancer Cells under Diabetic Conditions: Role of Regulatory Kinases—Rho Kinase and Focal Adhesion Kinase

**DOI:** 10.3390/cancers16183166

**Published:** 2024-09-15

**Authors:** Diganta Dutta, Matthew Ziemke, Payton Sindelar, Hernan Vargas, Jung Yul Lim, Surabhi Chandra

**Affiliations:** 1 Department of Physics, Astronomy and Engineering, University of Nebraska at Kearney, Kearney, NE 68849, USA; 2 Department of Biology, University of Nebraska at Kearney, Kearney, NE 68849, USAvargash@lopers.unk.edu (H.V.); 3 Department of Mechanical and Materials Engineering, University of Nebraska-Lincoln, Lincoln, NE 68588, USA

**Keywords:** atomic force microscopy, breast cancer cells, diabetes, Rho kinase, focal adhesion kinase, cytoarchitecture, elasticity

## Abstract

**Simple Summary:**

Diabetes rapidly advances the growth and spread of breast cancer. This study explores how high-glucose conditions in diabetes affect the structure of cancer cells, which makes them more aggressive and more likely to spread to other parts of the body. This is partly due to the involvement of two proteins associated with cytoskeletal structure, Rho kinase (ROCK) and focal adhesion kinase (FAK), which are important for cell movement. Cell shape and stiffness were studied using advanced microscopic techniques in cancer cells and normal cells exposed to varying glucose levels. It was found that cancer levels made the cancer cells less stiff and more likely to move, which can lead to the cancer spreading. However, when the ROCK or FAK proteins were blocked, these harmful changes were lessened.

**Abstract:**

Diabetes greatly reduces the survival rates in breast cancer patients due to chemoresistance and metastasis. Reorganization of the cytoskeleton is crucial to cell migration and metastasis. Regulatory cytoskeletal protein kinases such as the Rho kinase (ROCK) and focal adhesion kinase (FAK) play a key role in cell mobility and have been shown to be affected in cancer. It is hypothesized that diabetes/high-glucose conditions alter the cytoskeletal structure and, thus, the elasticity of breast cancer cells through the ROCK and FAK pathway, which can cause rapid metastasis of cancer. The aim of the study was to investigate the role of potential mediators that affect the morphology of cancer cells in diabetes, thus leading to aggressive cancer. Breast cancer cells (MDA-MB-231 and MCF-7) were treated with 5 mM glucose (low glucose) or 25 mM glucose (high glucose) in the presence of Rho kinase inhibitor (Y-27632, 10 mM) or FAK inhibitor (10 mM). Cell morphology and elasticity were monitored using atomic force microscopy (AFM), and actin staining was performed by fluorescence microscopy. For comparative study, normal mammary breast epithelial cells (MCF-10A) were used. It was observed that high-glucose treatments modified the cytoskeleton of the cells, as observed through AFM and fluorescence microscopy, and significantly reduced the elasticity of the cells. Blocking the ROCK or FAK pathway diminished the high-glucose effects. These changes were more evident in the breast cancer cells as compared to the normal cells. This study improves the knowledge on the cytoarchitecture properties of diabetic breast cancer cells and provides potential pathways that can be targeted to prevent such effects.

## 1. Introduction

Diabetes and cancer are among two of the leading causes of death in the United States [1]. The American Cancer Society estimates that around 2 million new cases of cancer will be diagnosed in the year 2024 [2]. Diabetes has been observed to be rising at a rapid rate, with around 11.3% of the United States’ population living with the condition [3]. The coexistence of diabetes and cancer has been linked to a decrease in cancer prognosis, an increase in cancer metastasis, and an increase in mortality rates in patients [4,5]. Cancers of the liver, colon, pancreas, and breasts have all been shown to have an increased number of malignancies in patients in a diabetic state [6].

The progression of cancer relies heavily on the ability of malignant cells to migrate and metastasize to other parts of the body. A key component to the metastatic potential of the cancer cells is in their epithelial-to-mesenchymal transition, which gives them the ability to dissociate from the primary tumor, intravasate the blood vessels, extravasate at a distal site, and establish a secondary tumor [7]. There are currently around 140,000 patients living with metastatic breast cancer, which is ranked second after cancer of the respiratory system, according to National Cancer Institute data [8,9].

Cytoarchitecture is a key cellular feature responsible in the metastatic process as its integrated regulatory proteins, such as focal adhesion kinase (FAK) and rho kinase (ROCK), function in cell motility and adhesion. FAK is a non-receptor tyrosine kinase that plays a central mediatory role in integrin signaling and cytoskeletal rearrangement; thus, it is significantly involved with dysregulated cellular processes underlying diabetic complications such as impaired wound healing, nephropathy, and cardiovascular complications. FAK’s involvement in cancer progression is well-documented, with its overexpression and hyperactivation frequently observed across various malignancies [10]. ROCK signaling plays a key role in podocyte formation by modulating the reorganization of microtubules and actin [11]. ROCK has been shown to be elevated in several cancers including those of the stomach, colon, bladder, and malignant melanomas [12]. By virtue of their ability to modulate signaling pathways involved in cell survival, migration, and epithelial-mesenchymal transition (EMT), FAK and ROCK serve a key role in cancer cell behavior and metastatic dissemination.

Although the distinct pathophysiological contexts of diabetes and cancer seem to be disparate, emerging evidence defines significant overlaps in the molecular pathways driving both conditions. Hyperglycemia, hyperinsulinemia, and chronic inflammation, which are all hallmarks of diabetes, create a tumor-promoting environment that can enhance the proliferation, survival, and metastasis of cancer cells. Certain mediators including diabetes-induced oxidative stress and alterations in the extracellular matrix can disrupt cell cycle regulation and apoptosis, contributing to cancer progression. In this manuscript, the dual role of FAK and ROCK as molecular switches governing cellular behavior in the context of diabetic breast cancer was explored. The hypothesis of the study is that ROCK and FAK are involved in the modified cytoarchitecture of breast cancer cells under hyperglycemic conditions. The aim of the study is to investigate the role of potential mediators that affect the morphology of cancer cells in diabetes, thus leading to aggressive conditions. Modification of cellular architecture and change in elasticity was determined using atomic force microscopy (AFM), while actin staining in cells was visualized using fluorescence microscopy. AFM is a very high-resolution, probe-based microscopy used for imaging nano-scale sample surfaces. AFM applications range from biological tissue imaging to material structure examination to nanotechnology. This tool is also used for force measurements.

By elucidating the shared signaling cascades and molecular mechanisms affecting breast cancer proliferation and metastasis in diabetic states, potential therapeutic avenues targeting FAK and ROCK for the management of these pathologies can be determined.

## 2. Materials and Methods

### 2.1. Reagents and Supplies

Dulbecco’s Modified Eagle Medium (DMEM) (catalog #11965092), glucose-free phenol red-free DMEM (catalog #A1443001), horse serum (catalog #16050122), penicillin/streptomycin (catalog #15070063), GlutaMAX (catalog #35050061), PrestoBlue^®^ cell viability reagent (catalog #A13262), and epidermal growth factor (EGF, catalog #AF-100-15) were obtained from Thermo Fisher Scientific (Rockford, IL, USA). All cell lines—MDA-MB-231 (catalog #HTB-26), MCF-7 (catalog #HTB-22) and MCF-10A (catalog #CRL-10317), and fetal bovine serum (FBS, catalog #30-2020)—were obtained from American Type Culture Collection (Manassas, VA, USA). Glucose (catalog #G7021), insulin (catalog #I1882), hydrocortisone (catalog #H0888), and cholera toxin (catalog #C8052) were obtained from Millipore Sigma (Milwaukee, WI, USA). The inhibitors, focal adhesion kinase inhibitor (FAK Inhibitor 14, SML0837) and Rho kinase inhibitor (Y-27632, catalog #Y0503), were also obtained from Millipore Sigma (Milwaukee, WI, USA).

### 2.2. Cell Culture Conditions

The cell lines used in this study—triple negative breast cancer cells (TNBC; MDA-MB-231), hormone receptor positive breast cancer cells (MCF-7), and normal mammary epithelial cells (MCF-10A)—were all cultured at 37 °C in a humidified atmosphere with 5% CO_2_. For maintenance and subculture, breast cancer cells were grown in DMEM supplemented with 5% FBS, 100 IU/mL penicillin, and 100 μg/mL streptomycin. MCF-10A cells were grown in DMEM/F12 supplemented with 5% horse serum, 20 ng/mL EGF, 0.5 µg/mL hydrocortisone, 100 ng/mL cholera toxin, 10 μg/mL insulin, 100 IU/mL penicillin, and 100 μg/mL streptomycin. 

For treatment, after cells reached their assay-specific confluency (80% for AFM and 50% for fluorescence microscopy), they were treated for 24 h in glucose-free, phenol red-free DMEM media supplemented with varying concentrations of glucose (5 mM or 25 mM), 1% FBS or 1% horse serum, 1% GlutaMAX, 100 IU/mL penicillin, and 100 µg/mL streptomycin. Glucose-free media allow for manipulation of glucose concentrations, which were varied from low (5 mM glucose) to high (25 mM glucose) for this study. In addition, phenol red-free media allows for better visualization of cells under microscope. Concentration of FBS was reduced to prevent potential interactions of serum binding proteins with the inhibitors during treatment. Focal adhesion kinase (FAK), indicated in this report by ‘F’, and rho kinase (ROCK), indicated by ‘Y’, were introduced in these treatment solutions as a part of this study. 

### 2.3. Atomic Force Microscopy

Nano-indentation experiments were performed with the help of AFM in this study, following methods used in previous studies [13,14,15,16,17,18]. The dimensions of cells were scanned using an Oxford Instruments MFP-3D atomic force microscope (AFM), utilizing Bruker MSNL silicon nitride probes possessing multiple cantilevers supplied by MultiMode 8 with NanoScope V controller (Bruker Nano, Santa Barbara, CA). AFM can work in contact and noncontact mode. Contact mode was used for cell imaging. MSNL-F probes were used for force measurements. We used MSNL-E probes (Bruker, CA) with nominal spring constant 0.1 N/m. The measured K was approximately 0.15 N/m in our experiments. AFM probe was calibrated with polydimethylsiloxane (PDMS), a sample known to have a modulus of elasticity of 2.5 MPa. We used Peak Force Quantitative Nanomechanical Mapping (PF QNM) probe, which does not imply resonance oscillation to the cantilever. The Sneddon model was used for calculating modulus of elasticity [19]. In Equation (1), *F* represents force, *E* represents modulus of elasticity, *ν* represents the Poisson ratio, *α* is the half-angle of the intender, and *δ* represents indentation depth. In this work, the Poisson ratio is 0.5 and angle is 53 degrees. In this model, force is the function of indentation depth. When force is increased over the sample, the sample/cells indent by a power of 2. AFM was used to image three different types of cells, and nano-indentation was performed over these cells.

(1) $$F=\frac{2}{\pi }\frac{E}{\left(1-\nu^{2}\right)}tan\left(\alpha \right)\delta^{2}$$

### 2.4. General Data Acquisition

Each image collected ~16 K (128 × 128) force curves for analysis. There are 40 to 83 force curves analyzed from these data points. Every force curve has a loading–unloading curve. The magnitude of the AFM force ranges from 1 nN to 2 nN. The loading force was used to calculate the modulus of elasticity. In this work, the atomic force microscopy probe approached and retracted at a rate of 3–6 µm/sec. Multiple cells were measured with changing parameters for each sample via force map imaging. Where an arrow is present in the figure imagery, we are indicating the location of where measurements were taken in the center of cells. The standard error for each condition was used to determine the statistical significance of each value. FemtoScan was utilized to record height and roughness data [20]. In all experiments, conditions were controlled such that force and angle of application remained constant so that the elasticity of the cells post-treatment would yield an indentation depth, represented by *δ* in the Sneddon model, to be recorded. This affords a controlled experiment to see how each cell group’s elasticity changed with treatment.

### 2.5. Fluorescence Microscopy

Cells were plated in tissue-culture-treated chambered glass slides (Fisher Scientific, Pittsburgh, PA, catalog #12-565-7) and treated with varying concentrations of glucose in presence of the FAK or ROCK inhibitors. After the incubation, cells were washed with PBS, fixed in 4% paraformaldehyde for 15 min, washed again with PBS, and then permeabilized using 0.5% triton X-100. Following permeabilization, cells were washed again and then incubated with actin red (Thermofisher, Waltham, MA, USA, catalog #R37112) at a concentration of 2 drops/mL media for 30 min. DAPI (Thermofisher, Waltham, MA, USA, catalog #P36931), at a concentration of one drop/mL, media were used for nuclear staining. After removal of the dye and washing with PBS, cells were visualized using fluorescence microscope (Nikon Inverted TE2000 Fluorescent Microscope, Melville, NY, USA) at 545 nm excitation and 565 nm emission for actin staining and 359 nm excitation (UV light) and 461 nm emission for DAPI staining.

### 2.6. Statistical Analysis 

All values obtained were expressed as mean ± SEM. Results shown were analyzed with Graph Pad software (Prism 5.0) [21]. Statistical comparison between more than two different groups was performed using one-way ANOVA followed by Tukey’s test. Differences were statistically significant at *p* < 0.05.

## 3. Results

### 3.1. Effect of High Glucose on the Topography of MDA-MB-231 Cells

**Figure 1:**
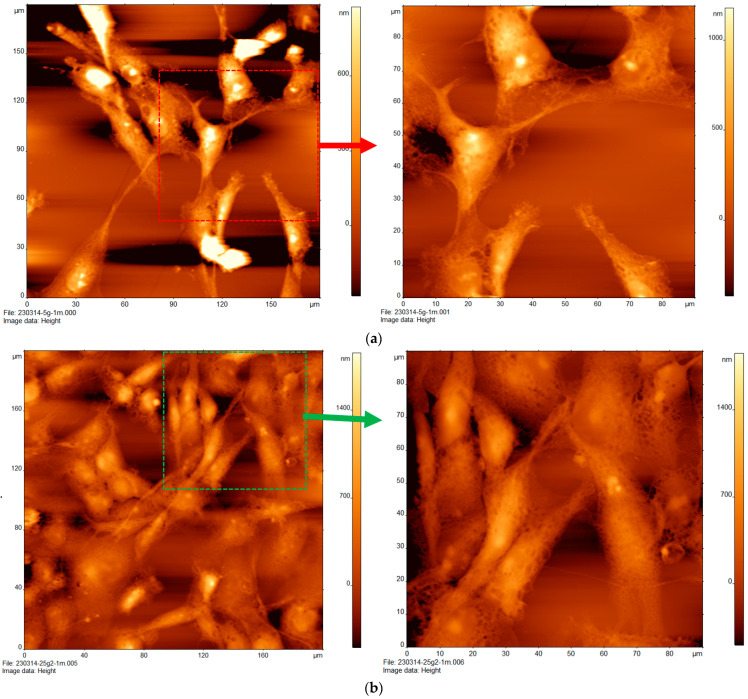
Atomic force microscopy of triple negative breast cancer cells (MDA-MB-231) treated with varying concentrations of glucose. Cells treated with (**a**) low glucose 5 mM and (**b**) high glucose 25 mM.

The study used two concentrations of glucose. The low-glucose treatment had a concentration of 5 mM glucose and is referred to as 5G. The high-glucose treatment had a concertation of 25 mM glucose and is referred to as 25G. Figure 1a shows the AFM images of MDA-MB-231 cells treated with low glucose (5G). Roughly ten cells are present within a 180 × 180-micron field of view. The cells are irregular ovoid shapes interconnected to one another by thin fibrous organelles. The topography of the cells ranges from dark orange (0 µm) to yellow–white (900 µm). A subtle texture is visible on each cell but they are otherwise relatively smooth. The dotted red square, highlighted to the right, shows the cancer cells and AFM scan range of 0–90 µm. Greater magnification indicates that the connective organelles have a fibrous, net-like structure. The cells have a slight texture to them but are otherwise somewhat smooth.

Figure 1b shows the AFM images of MDA-MB-231 treated with high glucose (25G). AFM was used to scan at a magnification of 180 × 180 µm, revealing that the cells are distinct in their tight groupings and are slenderer in appearance. The connective features between cells have a less dramatic taper to them. There are roughly 20–25 individual cells packed together in small groupings of 3–5 cells. Their heights range from 0 to 1500 nm. The green dotted box in the upper right corner of the image indicates the location of the right panel, where a small cluster of 11 cells at an AFM scan range of 0–90 microns is observed. Here, a smooth but sponge-like texture of the connective features between the cells is present.

### 3.2. Effect of Rho Kinase Inhibitor in Combination with Glucose Treatments on the Topography of MDA-MB-231 Cells

**Figure 2:**
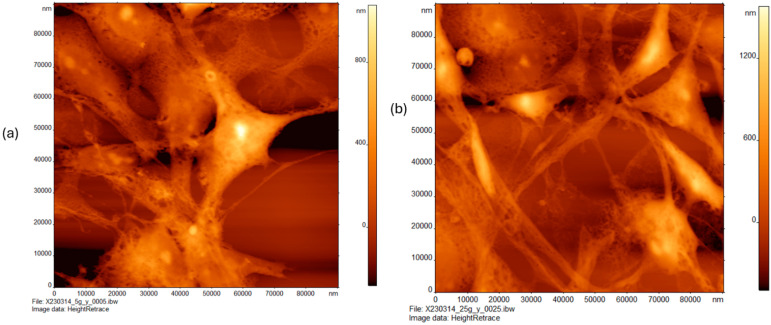
Atomic force microscopy of triple negative breast cancer cells (MDA-MB-231) treated with varying concentrations of glucose in presence of Rho kinase inhibitor Y-27,632 (Y, 10 mM) for 24 h. Cells treated with (**a**) low glucose (5 mM) in combination with Y (10 mM) and (**b**) high glucose (25 mM) in combination with Y (10 mM).

Figure 2a depicts the AFM images of MDA-MB-231 cells treated with low glucose (5G) in combination with the Rho kinase inhibitor (Y), presented in a scan area of 0 × 90 µm. This is a tight grouping of cells resembling that of other specimens except for their proximity and thinner connective structures. These cells also appear to be slightly more featured with distinct spots present on the cells. These heights read from 0 to 1 µm.

Figure 2b shows the AFM images of MDA-MB-231 cells treated with high glucose (25G) in combination with the Rho kinase inhibitor (Y), shown at a scan range of 0–90 µm. These cells are more triangular with a subtle taper between the cell and connective feature and a smooth but slightly featured, sponge-like surface. These cells are more widely spaced. Height ranges from 0 to about 1400 nm.

### 3.3. Effect of Focal Adhesion Kinase (FAK, F) Inhibitor in Combination with Glucose Treatments on Topography of MDA-MB-231 Cells

**Figure 3:**
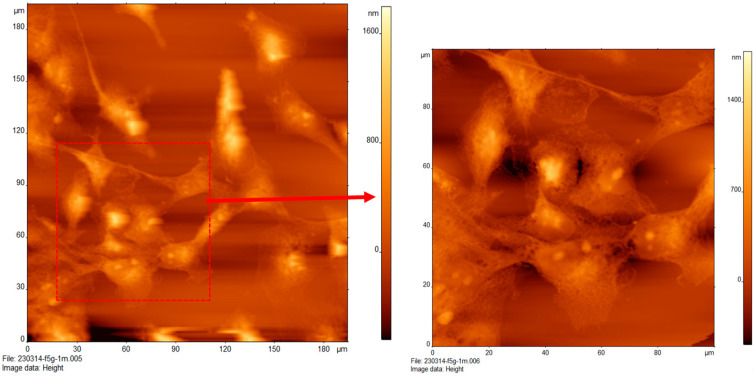
Atomic force microscopy of triple negative breast cancer cells (MDA-MB-231) treated with varying glucose in presence of FAK inhibitor (F, 10 mM) for 24 h. Cells treated with low glucose (5 mM) in combination with F (10 mM). The red dotted square highlights a region further magnified to the right.

Figure 3 shows the AFM images of MDA-MB-231 treated with low glucose (5G) in combination with FAK inhibitor (F). A network of cells is displayed when AFM scans at 190 × 190 µm. Roughly ten cells with somewhat smooth surfaces are connected by network-like organelles. The cells’ height reads between roughly 400 and 1600 nm. The red dotted square highlights the area under greater magnification. At this magnification of 0–90 µm, the texture of the cells is more subtle. Webbed features radiate from each cell, becoming the connective features discussed previously. The cells themselves appear smoother than the connective features. 

### 3.4. Effect of High Glucose on the Topography of MCF-7 Cells

**Figure 4:**
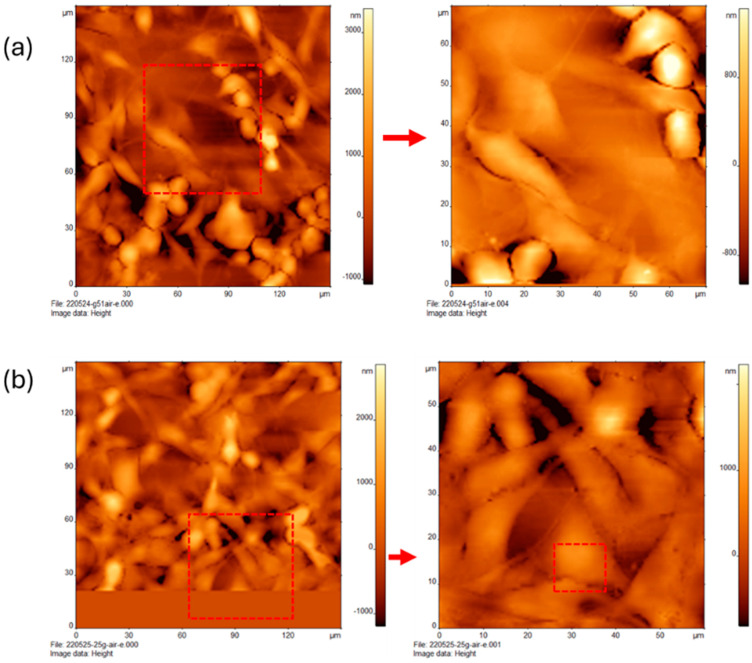
Atomic force microscopy of breast cancer cells (MCF-7) treated with varying concentrations of glucose for 24 h. Cells treated with (**a**) low glucose 5 mM and (**b**) high glucose 25 mM.

The AFM images of MCF-7 cells treated with low glucose (5G) are shown in Figure 4a. These cells are smaller than the MDA-MB-231 cells, at 10–20 microns across. They are grouped in clusters of 3–10 cells and do not display much texture. The connective features between the cells are less frequent than in other samples and heights range from 3 to 4 µm. The highlighted square indicates the region of cells presented at greater magnification on the right and the AFM scan ranges from 0 to 70 microns. There is some smoothness to the cells and some of the connective features observed here.

The AFM images of MCF-7 cells treated with high glucose (25G) are shown in Figure 4b and the AFM scan region of 0–150 microns. Here, the tightly packed cells are ovoid in shape with smooth transitions to connective features. The red dotted square highlights the region under the zoom-in section presented at 0–60 µm. The cells are very smooth and range in height from 1 to 1.5 µm.

### 3.5. Effect of Rho Kinase Inhibitor in Combination with Glucose Treatments on the Topography of MCF-7 Cells

**Figure 5:**
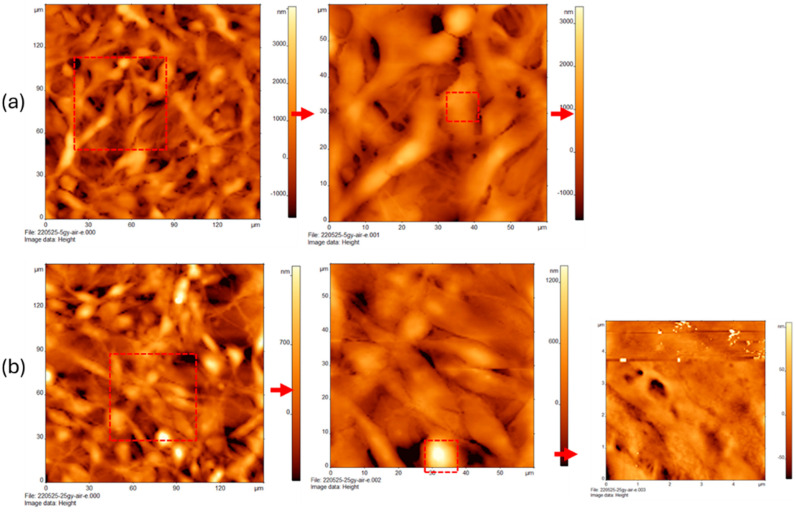
Atomic force microscopy of breast cancer cells (MCF-7) treated with varying concentrations of glucose in presence of Rho kinase inhibitor Y-27632 (Y, 10 mM) for 24 h. Cells treated with (**a**) low glucose (5 mM) in combination with Y (10 mM) and (**b**) high glucose (25 mM) in combination with Y (10 mM).

The AFM images of MCF-7 treated with low glucose (5G) in combination with Rho kinase inhibitor (Y) are shown in Figure 5a, with tightly grouped cells that form a consistent network of elongated cells. The connective features in these cells are not as distinctly different from the cells themselves as in previous examples. The cells are more elongated than ovoid. The red dotted square indicates the area of focus in the right panel, with a scan range from 0 to 60 microns. The heights of the cells here appear to be around 3 µm. The surface texture is very smooth.

The three windows in Figure 5b present the AFM images of MCF-7 cells treated with high glucose (25G) in combination with Rho kinase inhibitor (Y). The left window presents a region of cells at a scan range of 0–150 microns. Here, the cells are consistently spread across the field of view with a few sections of more open space. The cells are irregular, ovoid, and have thinner connective features. The red square indicates the region of interest in the middle with the cells having a very smooth texture, and heights range from 600 to 1200 nm. The rightmost figure shows a zoomed-in region from 0 to 60 µm. Cells are roughly 1.2 µm across, with little surface variation visible.

### 3.6. Effect of High Glucose on the Topography of MCF-10A Cells

**Figure 6:**
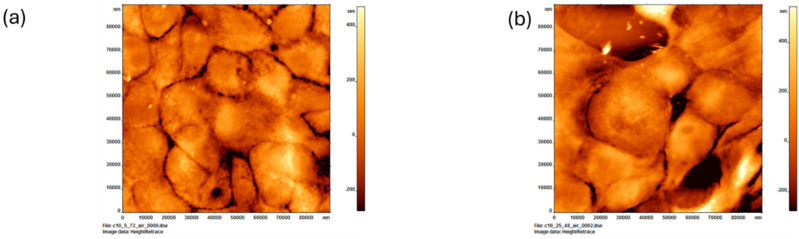
Atomic force microscopy of normal mammary epithelial cells (MCF-10A) treated with varying concentrations of glucose for 24 h. Cells treated with (**a**) low glucose 5 mM and (**b**) high glucose 25 mM.

Figure 6a shows a single AFM image of MCF-10A treated with low glucose (5G). The scan range is 100 µm × 100 µm. The height of the cells is about 600 nm, and the size of the cells is 20 µm. These cells are closely packed with one another. Images were taken in the air.

Figure 6b depicts a single AFM image of MCF-10A cells treated with high glucose (25G). It displays a region of cells magnified 100 × 100 µm, as in Figure 6a. These cells are bulbous and interconnected with connective features that act more as an extension of the round healthy cells rather than round tightly packed cells, as in Figure 6a. It is good to note that these healthy cells do not display the filamentous network of interconnected features as in cancerous cells, particularly that of the MCF-7 line. This structural contrast to cancerous cell networks was documented by Wang et al. in a 2016 study [22]. The cells range from 15 µm to 32 µm across. The surface texture is primarily smooth with little variation across the surface. Heights range from 400 nm to 500 nm. 

### 3.7. Changes in Modulus of Elasticity with Glucose and Cytoskeletal Inhibition (Rho Kinase, FAK) in MDA-MB-231 Cells

**Figure 7:**
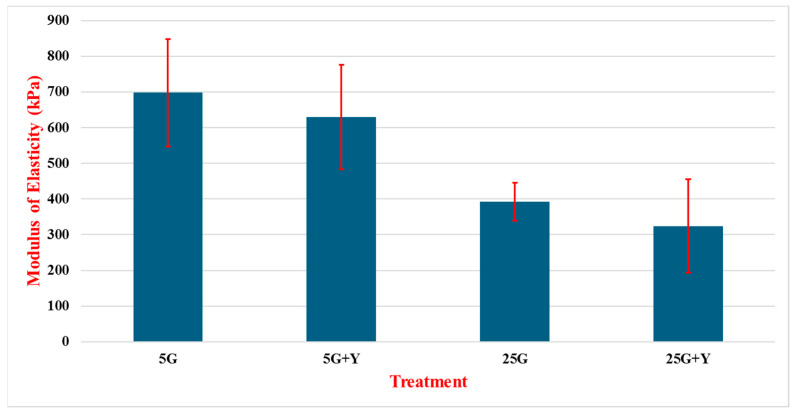
Modulus of elasticity of MDA-MB-231 cells treated with varying concentrations of glucose in the presence of the Y compound. Cells were treated for 24 h with low glucose (5 mM, 5G), high glucose (25 mM, 25G), low glucose with inhibitor (5G + Y), and high glucose with inhibitor (25G + Y). N = 28~75.

**Figure 8:**
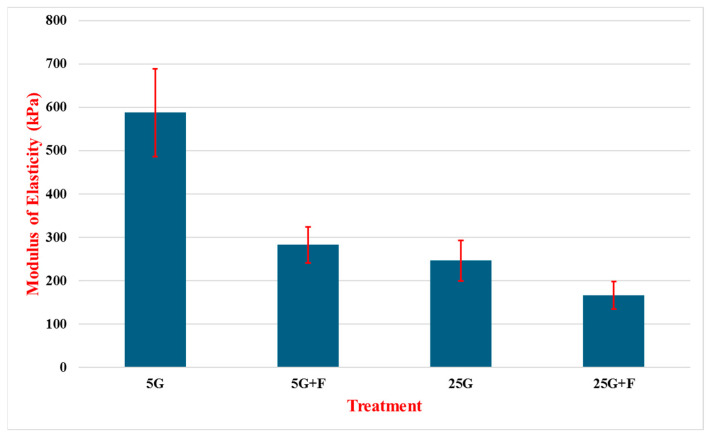
Modulus of elasticity of MDA-MB-231 cells treated with varying concentrations of glucose in the presence of the F compound. Cells were treated for 24 h with low glucose (5 mM, 5G), high glucose (25 mM, 25G), low glucose with inhibitor (5G + F), and high glucose with inhibitor (25G + F). N = 50~83.

Figure 7 shows the modulus of elasticity (MOE) of cells MDA-MB-231 treated with glucose and Rho kinase inhibitor (Y). Tests on cell line MDA-MB-231 provided notably different results with the addition of +Y over +F in the glucose treatments. The 5G treatment provided the highest MOE for the cell line at 698.2 kPa, with a standard deviation of 150.5 kPa between 75 tests. In this case, the 5G + Y treatment produced higher values than in the +F treatments at 629.5 kPa, with a standard deviation of 146.6 kPa between 75 experiments (Figure 8). The 25G and 25G + Y treatments on 231 were lower than the previous two treatments, with the value at 392.1 kPa presenting a standard deviation of 53.3 over 28 experiments and 324.3 kPa showing a standard deviation of 131.4 over 30 tests. 

Figure 8 shows the modulus of elasticity of MDA-MB-231 cells treated with glucose and FAK inhibitor (F). In the 5G treatment, cell line MDA-MB-231 demonstrated an elasticity value of 587.6 kPa with a standard deviation of 101.6 kPa across 50 tests. Successive tests produced notably lower MOE values. The 5G + F treatment resulted in a tested MOE of 282.7 kPa with a standard deviation of 41.2 kPa over 50 tests, just over half the value of the elasticity of cells treated with 5G. The 25G and 25G + F treatments produced lower E values at 246.5 kPa with a standard deviation of 46.9 over 52 tests and at 166.5 kPa with a standard deviation of 31.7 kPa over 83 tests. It is important to note in this graph the dramatic decrease in MOE of the MDA-MB-231 cells under the 5G + F treatment over the 5G + Y treatment, as seen in Figure 7.

### 3.8. Changes in Modulus of Elasticity with Glucose and FAK Inhibition in MCF-7 Cells

**Figure 9:**
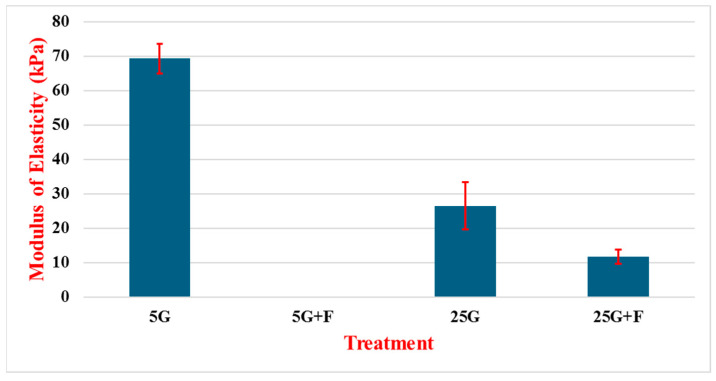
Modulus of elasticity of MCF-7 cells treated with varying concentrations of glucose in the presence of the F compound. Cells were treated for 24 h with low glucose (5 mM, 5G), high glucose (25 mM, 25G), low glucose with inhibitor (5G + F), and high glucose with inhibitor (25G + F). N = 40~50.

Figure 9 shows the modulus of elasticity of MCF-7 cells treated with glucose and FAK inhibitor (F). The 5G treatment resulted in an MOE of 69.3 kPa and standard deviation of 4.3 kPa over 50 experiments. Successive experiments produced lower MOE value results, with 25G and 25G + F giving a modulus of 26.5 kPa with a standard deviation of 6.8 kPa over 40 experiments and 11.7 kPa with a standard deviation of 2 kPa over 40 experiments. Across the three treatments, there is a correlation between lower glucose levels and increased cell stiffness, as the 5G treatment demonstrates the highest MOE value of all three groups at 2.6 times stiffer than the 25G treatment and almost 6 times stiffer than the cells treated with 25G + F. Results of the cell response to the 5G + F treatment did not return reliable enough data to include in this study.

### 3.9. Changes in Modulus of Elasticity with Glucose and Cytoskeletal Inhibition (Rho Kinase, FAK) in MCF-10A Cells

**Figure 10:**
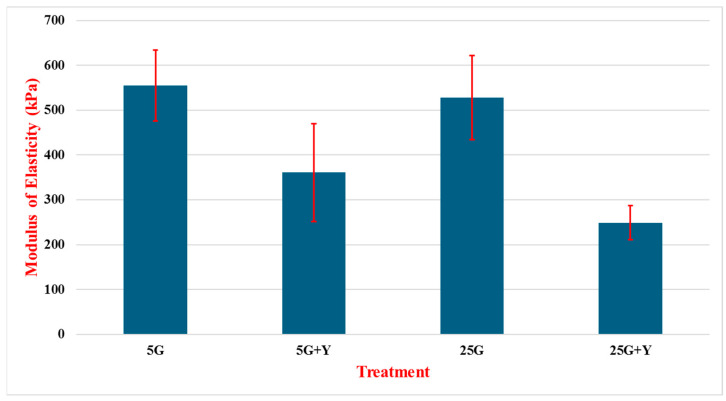
Modulus of elasticity of MCF-10A cells treated with varying concentrations of glucose in the presence of the Y compound. Cells were treated for 24 h with low glucose (5 mM, 5G), high glucose (25 mM, 25G), low glucose with inhibitor (5G + Y), and high glucose with inhibitor (25G + Y). N = 78~38.

Figure 10 shows the modulus of elasticity of MCF-10A cells treated with glucose and Rho kinase inhibitor (+Y). With the 5G treatment, the cells demonstrated an MOE of 555 kPa on average, with a standard deviation of 79 kPa across 78 experiments. The 5G + Y experiments resulted an average of 361 kPa with a standard deviation of 109 across 78 experiments. The +Y formula produces a clearly lower modulus of elasticity in the cells, as the 5G solution produced over 1.5 times greater MOE values on average. The 25G treatment produced the second highest MOE value at 528 kPa with a standard deviation of 94 kPa, and the 25 G +Y saw an average E value of 249 kPa with a standard deviation of 38 kPa across 40 experiments. This gives us a relationship of the 25G treatment producing a roughly two times higher MOE value than the 25G + Y. Though the 25G treatment saw a slightly lower E value than did the 5G, the addition of +Y had less of an effect on the elasticity of cells in the experiment than that of the 5G and 5G + Y if we compare the 1.5 times and 2 times greater in the non-Y treatment mentioned before. Despite the obvious difference of 27 kPa between the 5G and 25G experiments, each produced notably higher MOE values than the experiments with ‘+Y’.

### 3.10. Actin Staining in MDA-MB-231 Cells upon Treatment with Glucose and FAK Inhibitor

**Figure 11:**
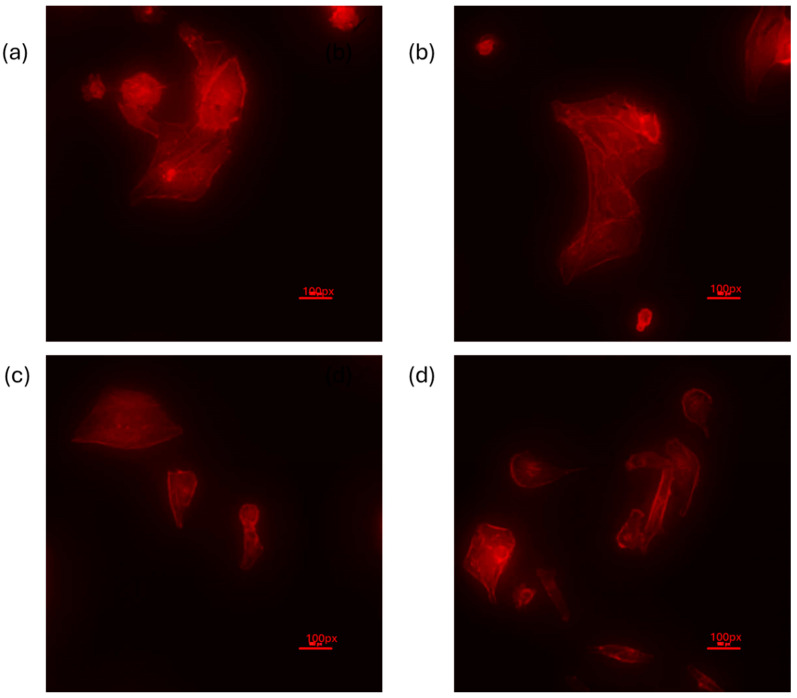
Actin staining of MDA-MB-231 cells treated with varying concentrations of glucose in the presence of the F compound. Cells were treated for 24 h with (**a**) low glucose (5 mM, 5G), (**b**) high glucose (25 mM, 25G), (**c**) low glucose with inhibitor (5G + F), and (**d**) high glucose with inhibitor (25G + F).

Fluorescence staining of actin in the MDA-MB-231 cells revealed a clear actin structure in the cells treated with low glucose, 5G, but the staining was very faint in 25G (Figure 11b) and almost negligent in the inhibitor treatments (Figure 11c,d). Actin fibers show a linear arrangement in the 5G treatment (Figure 11a) but show a more diffuse pattern in high glucose (25G) as well as in the cells treated with glucose and the FAK inhibitor (Figure 11b–d).

## 4. Discussion

The nature of a cancerous cell’s cytoskeleton plays a crucial role in how it metastasizes [23]. The cytoskeleton comprises a network of filaments (actin microfilaments, microtubules, and intermediate filaments), arranged in complex meshes that provide cells their shape and, therefore, stiffness [24]. The mechanics of a cell indicate the state of its cytoarchitecture and how the cytoarchitecture then dictates cell function [25,26]. We can analyze these mechanics using the modulus of elasticity to compare resulting changes following treatments, as we have performed in this study. This lower MOE allows cancerous cells to more easily migrate in the body. Our findings suggest that high-glucose environments increase this low MOE and could create an environment where cancers can become more aggressive in their metastasis. Research into breast cancer cell cytoskeletal characteristics by Hadaryan et al. reported that cancerous cells have a roughly 3.5 times lower modulus of elasticity (MOE) than that of healthy cells [27], while similar work by Li et al. found a modulus of elasticity of 1.4 to 1.8 times lower [28]. Work by Koreyam et al. showed how a lower Young’s modulus and lower adhesion is characteristic of cancerous MCF-7 cells when compared to MCF-10A cells [29]. Additionally, studies using alternate methods of analyzing the cytoarchitecture of cancerous cells with regard to their MOE, such as optical deformability, indicate a notably softer cell structure in contrast to healthy cells [30]. Research into how culture conditions might alter MOE in both MDA-MB-231 and healthy MCF-10A cells by Nikkhah et al. indicated that while both serum percentages and growth mediums can have an effect on the stiffness of cultured cells, the MOE between healthy and cancerous cells remained softer in MDA-MB-231, consistent with the hypothesis and results of this study [31]. The significance of the behavior of these cells, as these findings suggest, is that because of the cells’ lower adhesion, these cancerous cells are more likely to break away from their native tumor and invade surrounding tissues, leading to metastasis.

In this study, we focused on measuring the modulus of elasticity and surface characteristics of two different breast cancer cells (MDA-MB-231 and MCF-7) after treatment. Treatments involved varying the glucose concentrations of solutions containing either no additive or the addition of cytoskeletal inhibitors Rho kinase (Y) or FAK (F). In general, lower glucose levels resulted in higher E values, translating to greater stiffness in tested cells. Conversely, higher glucose concentrations in testing correlated to a lowering of Young’s modulus of elasticity. Work by Zou et al. suggested an inverse relationship between cell stiffness and malignancy in the MCF-7 and MDA-MB-23 cancer cells lines, where treatments high in glucose resulted in a lowered elasticity modulus [32]. This would suggest that high-glucose environments, such as that found in diabetic patients, could lead to softening of tissues and promote metastasis. Test results varied in terms of the change in Young’s modulus for each treatment. MCF-10A was less responsive to glucose concentrations relative to that of MCF-7 and MDA-MB-231. Referencing Figure 10, we see that the 5G solution yields the highest Young’s Modulus compared with the 25G solution, showing a difference of only 27 kPa between them. In the same tests, a glucose solution with the addition of Y greatly reduced Young’s modulus of the cells relative to treatments without it. It should be noted that the presence of Y in higher glucose concentrations (25G) had an even more dramatic effect on reducing the stiffness of the cells in the tests, suggesting that higher glucose levels in addition to Y lead to greater softening of cells rather than glucose treatments alone. Between the 5G + Y and 25G + Y treatments, there was a reduction in stiffness of 112 kPA over the 27 kPa difference noted above where solutions did not contain Y.

Subsequent testing on the two other cell lines displayed a more consistent pattern in cell response to treatments with regard to Young’s modulus. In the MCF-7 cells, there is a much more dramatic softening of the cells between 5G and 25G solutions with a total difference of about 44 kPa or roughly a 2.75 times reduction. The addition of F was performed only for the 25G solution, which softened the cells even further, resulting in an added drop of roughly 14 kPa from the 25G solution alone.

There is a broader set of results from the MDA-MB-231 cells as there was testing with both the F and Y additives. While the exact numbers obtained with testing varied between the tests, each set clearly follows a similar pattern, where 5G produced higher Young’s modulus values; followed by that with the additive; then the 25G solution; and, finally, the lowest value is given by 25G with the additive. In testing the solutions using F, there was a notable difference between 5G and 5G + F as the additive resulted in a 305 kPa difference in Young’s modulus value (587 to 282 kPa). The drop in stiffness was most pronounced between these two tests within this data set, as the difference between 5G + F and 25G was only about 44 kPa (282 to 246 kPa). Not surprisingly, the 25G solution with the additive produced the lowest Young’s modulus value at 166.5 kPa. While the experiments using the Y additive demonstrated a similar trend in softening with an increase in glucose, this addition of Y resulted in more modest reductions in stiffness within each respective glucose concentration in the experiment. For example, in the 5G treatment, the Young’s modulus was reduced by 69 kPa in 5G + Y tests and 68 kPa between 25G and 25G + Y.

A similar study was conducted by Mariafrancesca Cascione et al. on the structural changes occurring in the same MCF-7 and MDA-MB-231 breast cancer cells with the ROCK inhibitor Y-27532. Here, the team was not focused on the role of ROCK in a hyperglycemic environment but found that the cytoskeletal structures were indeed changed with inhibitor treatments. Interestingly, they found that cellular rigidity increased with treatments of the inhibitor [33]. In this case, the team used Young’s modulus of elasticity as a gauge for stiffness. In contrast, our study found an overall trend in both FAK and ROCK treatments softening cellular structure in the presence of glucose, which may illustrate the unique nature of the hyperglycemic state on the metastasis of cancers.

ROCK and FAK are involved with actin formation [34]; hence, actin staining was performed to observe if there are any changes with high-glucose or -inhibitor treatments. It was clear that high glucose does affect the actin staining in MDA-MB-231 cells, with cells showing a diffuse pattern, and further inhibition of FAK markedly abrogated actin staining with very faint pictures. While several mediators might be involved with regulating cytoskeletal structure, the focus of this study was on some key molecules such as FAK and ROCK. In the future, the study will be extended to further investigate these pathways, and more cancer cell lines will be tested to determine common mediators, if any. The findings from this paper will serve as the basis for projecting the experiments to determine metastasis and invasion in cell culture and rodent models.

Moreover, microRNA-based gene therapy could be proposed to modulate the cytoskeletal pathways affected by hyperglycemia, offering a novel approach to counteract metastasis. Personalized medicine could benefit from these findings by tailoring treatment strategies based on the individual metabolic and molecular profiles of patients, particularly those with diabetes. Additionally, single-cell sequencing could be employed to understand the heterogeneity within TNBC tumors, identifying specific cell populations that respond differently to treatments and informing more precise therapeutic interventions (PMID: 34440380).

## 5. Conclusions

In conclusion, our results suggest that hyperglycemic conditions can significantly affect the cytoskeleton of breast cancer cells, which modulates their stiffness. This can be attributed to the epithelial to mesenchymal transition, further leading to the metastasis and invasiveness of these cells. Rho kinase and focal adhesion kinase inhibitors amplify the effects of high glucose, indicating that these cytoskeletal modulators should be regulated in cancer cells.

## Data Availability

All data are available upon request from the corresponding author.
